# Assessment of anxiety and depression levels using the HAM-A and HAM-D instruments in a group of patients with substance dependence disorders compared to a control group

**DOI:** 10.1192/j.eurpsy.2026.10818

**Published:** 2026-07-31

**Authors:** A. Saliu, L. Sinani, A. Pilika

**Affiliations:** 1 Psychiatry, Gjirokaster Hispital, Gjirokaster; 2 Psychiatry, Hospital of Mother Theresa Tirana, Tirana, Albania

## Abstract

**Introduction:**

Substance use disorders (SUDs) are chronic conditions with complex biological, psychological, and social consequences. During abstinence, many individuals continue to experience significant symptoms of anxiety and depression, which may be influenced by sociodemographic, hereditary, and clinical variables. This study examines the correlation between these factors and psychological symptoms in individuals diagnosed with SUDs, compared to a matched control group. Previous studies have identified neurobiological mechanisms involved in substance effects, particularly the role of exogenous agents on CNS structures (Murphy et al. BJU Int 2005; 95: 27–30).

**Objectives:**

To evaluate the levels of anxiety and depression among individuals with substance use disorders in abstinence (CVSA group) compared to a control group, using the Hamilton Rating Scales for Anxiety (HAM-A) and Depression (HAM-D).

**Methods:**

A cross-sectional, retrospective, quantitative, and descriptive study was conducted at the Toxicology and Addiction Department of the University Hospital Center “Mother Teresa” from October 2021 to February 2022. The sample included 109 male participants over the age of 18: 69 (55%) in the CVSA group and 49 (45%) in the control group. Exclusion criteria included the presence of diagnosed mental disorders. Data were collected via clinical interviews and hospital records, focusing on sociodemographic information, substance use history, family history of mental illness or substance abuse, and completion of the HAM-A and HAM-D scales.

**Results:**

Sociodemographic variables, substance use history, and hereditary factors showed a statistically significant correlation with elevated anxiety and depression levels in the CVSA group. The analysis revealed that early onset, type, and method of substance use were associated with more severe psychological symptoms during abstinence.

**Image 1:**
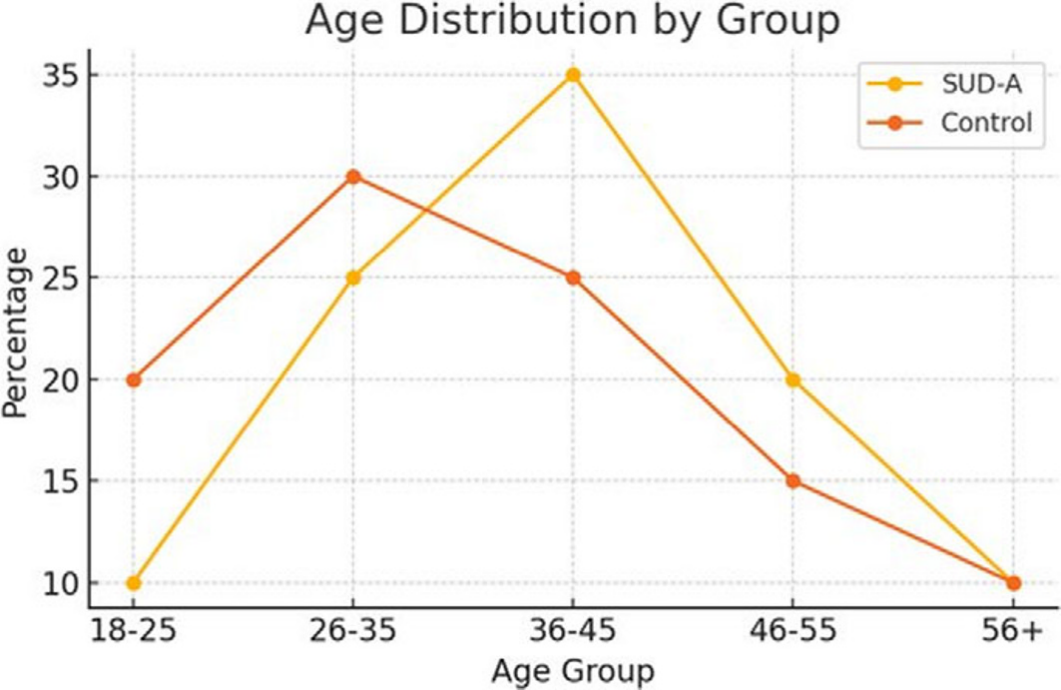
Image 1: Long description.

**Image 2:**
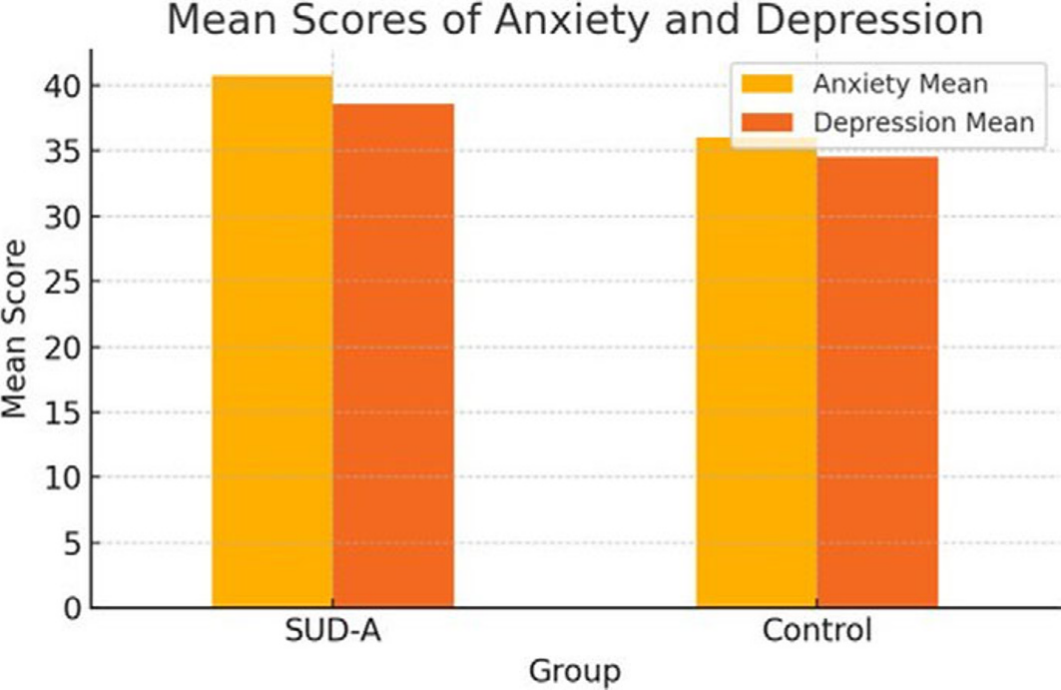

**Image 3:**
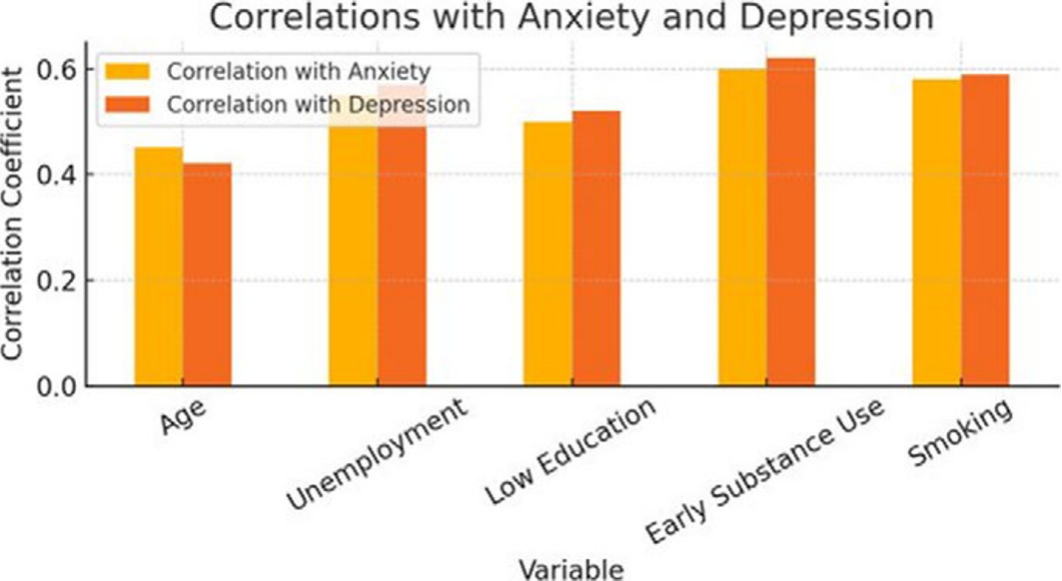
Image 3: Long description.

**Conclusions:**

The findings support the need for comprehensive psychiatric evaluations in individuals with SUDs, particularly during abstinence, to detect dual diagnoses early. This may improve treatment outcomes and reduce relapse risk.

**Disclosure of Interest:**

None Declared

## Long descriptions

### Image 1: Long description

The X axis is labeled Age Group with five categories: 18 dash 25, 26 dash 35, 36 dash 45, 46 dash 55, and 56 plus. The Y axis is labeled Percentage ranging from 10 to 35 in increments of 5.

Two lines are plotted:

* The S U D dash A group (yellow line) starts at 10 percent for age 18 dash 25, rises to 25 percent for 26 dash 35, reaches a peak of 35 percent at 36 dash 45, then drops to 20 percent for 46 dash 55 and ends at 10 percent for 56 plus.

* The Control group (orange line) starts higher at 20 percent for age 18 dash 25, peaks earlier at 30 percent for 26 dash 35, then steadily declines to 25 percent for 36 dash 45, 15 percent for 46 dash 55, and ends at 10 percent for 56 plus.

The two groups converge at the final data point of 10 percent for the 56 plus age group.

Navigate back to Image 1:.

### Image 3: Long description

The y-axis is labeled Correlation Coefficient and ranges from 0.0 to 0.6 with grid lines every 0.2 units. The x-axis is labeled Variable and contains five categories. A legend in the top-left indicates that yellow bars represent Correlation with Anxiety and orange bars represent Correlation with Depression.

Moving from left to right along the x-axis:

* Age: Anxiety is approximately 0.45 and Depression is approximately 0.42.

* Unemployment: Anxiety is approximately 0.55 and Depression is approximately 0.57.

* Low Education: Anxiety is approximately 0.50 and Depression is approximately 0.52.

* Early Substance Use: Anxiety is approximately 0.60 and Depression is approximately 0.62.

* Smoking: Anxiety is approximately 0.58 and Depression is approximately 0.59.

Overall, Early Substance Use shows the highest correlation for both conditions, while Age shows the lowest. In four out of five categories, the correlation with depression is slightly higher than the correlation with anxiety.

Navigate back to Image 3:.

